# Emerging role of tumor-related functional peptides encoded by lncRNA and circRNA

**DOI:** 10.1186/s12943-020-1147-3

**Published:** 2020-02-04

**Authors:** Pan Wu, Yongzhen Mo, Miao Peng, Ting Tang, Yu Zhong, Xiangying Deng, Fang Xiong, Can Guo, Xu Wu, Yong Li, Xiaoling Li, Guiyuan Li, Zhaoyang Zeng, Wei Xiong

**Affiliations:** 1 NHC Key Laboratory of Carcinogenesis and Hunan Key Laboratory of Translational Radiation Oncology, Hunan Cancer Hospital and The Affiliated Cancer Hospital of Xiangya School of Medicine, Central South University, Changsha, Hunan China; 2 Key Laboratory of Carcinogenesis and Cancer Invasion of the Chinese Ministry of Education, Cancer Research Institute, Central South University, Changsha, Hunan China; 3 Hunan Key Laboratory of Nonresolving Inflammation and Cancer, Disease Genome Research Center, the Third Xiangya Hospital, Central South University, Changsha, Hunan China; 4 Department of Medicine, Dan L Duncan Comprehensive Cancer Center, Baylor College of Medicine, Houston, Texas USA

**Keywords:** cancer, circRNA, lncRNA, peptide

## Abstract

Non-coding RNAs do not encode proteins and regulate various oncological processes. They are also important potential cancer diagnostic and prognostic biomarkers. Bioinformatics and translation omics have begun to elucidate the roles and modes of action of the functional peptides encoded by ncRNA. Here, recent advances in long non-coding RNA (lncRNA) and circular RNA (circRNA)-encoded small peptides are compiled and synthesized. We introduce both the computational and analytical methods used to forecast prospective ncRNAs encoding oncologically functional oligopeptides. We also present numerous specific lncRNA and circRNA-encoded proteins and their cancer-promoting or cancer-inhibiting molecular mechanisms. This information may expedite the discovery, development, and optimization of novel and efficacious cancer diagnostic, therapeutic, and prognostic protein-based tools derived from non-coding RNAs. The role of ncRNA-encoding functional peptides has promising application perspectives and potential challenges in cancer research. The aim of this review is to provide a theoretical basis and relevant references, which may promote the discovery of more functional peptides encoded by ncRNAs, and further develop novel anticancer therapeutic targets, as well as diagnostic and prognostic cancer markers.

## Background

The mammalian genome produces tens of thousands of non-coding transcripts during the transcription process. About 98% of the RNA in the human transcriptome is non-coding [1–4]. Non-coding RNA (ncRNA) is transcribed from the genome but not translated into protein. It controls various levels of gene expression during physiological and developmental processes, including epigenetic modification [5], transcription [6], RNA splicing [7], scaffold assembly [8], and others. NcRNAs have tissue-specific expression patterns and are potential biomarkers. Thus, they could serve as clinical diagnostic and prognostic indicators [9, 10]. Additionally, ncRNAs that were heretofore considered non-coding may, in fact, be able to encode small biologically active peptides [11–13]. Functional peptides are usually encoded by short open reading frames (sORFs) in ncRNAs [14–23]. NcRNAs can have one or more sORFs that can be translated into small peptides < 100 amino acids long. Previously, the traditional gene annotation process filtered out proteins < 100 amino acids by default and treated them as noise or false positives. Thus, they were always ignored [24]. However, as proteomics and translation technology have grown in popularity and increased in precision and accuracy, it was discovered that many ncRNAs are translatable [13, 25–28]. At present, it is recognized that long ncRNAs (lncRNAs) and circular RNAs (circRNAs) contain sORFs that can be translated into functional small peptides.

LncRNAs are generally defined as long RNA transcripts (>200 nucleotides) that do not encode proteins [29, 30]. The number of lncRNAs may exceed that of protein-coding transcripts. LncRNAs participate in the epigenetic regulation of gene expression [31, 32]. Several lncRNAs resemble mRNAs and can be transcribed, spliced, capped, and polyadenylated by RNA polymerase II-like protein-encoding pathways. These lncRNAs have tissue modification profiles, splicing signals, and exon/intron lengths similar to those of mRNAs [33–37]. In most cases, lncRNAs do not biochemically differ from mRNAs except that they lack reading frames encoding proteins. However, mass spectrometry, deep RNA sequencing, and other advanced molecular techniques have revealed that certain lncRNAs have non-random long sORFs [38–40], their exons are more highly conserved than those in protein-coding genes [41], they can interact with ribosomes [42, 43], and they could encode proteins. Mature microRNAs (miRNAs) are produced by the cleavage of primary transcripts (pri-miRNA) via a series of nucleases [44, 45]. Pri-miRNA is a special type of lncRNA hundreds to thousands of nucleotides long that is transcribed by RNA polymerase II-like protein-coding genes [46–49]. Therefore, pri-miRNA may also be able to encode proteins or peptides.

CircRNAs were recently discovered as ncRNAs with covalently closed structures. They regulate disease development and occurrence [50–52]. CircRNA is transcribed by RNA polymerase II without 5'-3' polarity or polyadenine tails. It has the same transcriptional efficiency as linear RNA [53–55]. CircRNA is hundreds to thousands of bases long and mainly consists of exons. Studies have shown that mammalian circRNAs are endogenous, abundant, conserved, and stable [56, 57]. CircRNAs are miRNA sponges [58–60] that control gene transcription. Moreover, the highly conserved ORFs in circRNAs encode functional peptides both in vivo and in vitro in a manner independent of the 5' cap structure, such as internal ribosome entry site (IRES) induction [61], promoting adenosine methylation (N6-methyladenosine; m6A) [62], rolling cycle amplification [63, 64], and others [65, 66]. As circRNA has a unique covalently closed structure, the ORFs therein circulate across the splicing site and even beyond its length. For this reason, it can also produce proteins > 100 amino acids long [67, 68].

The present review discusses current research progress in lncRNA- and circRNA-encoded proteins. It focuses on the fact that certain cancer-related lncRNAs and circRNAs encode functional small peptides that regulate biological processes and influence tumorigenesis, invasion, metastasis, and so on. The review also predicts and identifies potential ncRNAs that can encode functional small peptides.

### Prediction of ncRNA coding potential and identification of small peptides

In view of the increasing interest in ncRNA-encoded polypeptides, numerous prediction and experimental identification methods have been developed to determine the coding ability of ncRNAs. These include reading frame prediction, translation initiation component prediction, conservation analysis, and translation omics and proteomics, and others.

#### Open reading frame prediction

Open reading frames (ORFs) are nucleic acid sequences starting with ATG (or AUG in RNA) and continuing in three-base sets to a stop codon [69]. The length of sORFs is usually < 300 nt. Calculations [70] and ribosome analyses [43] have disclosed that thousands of unannotated ORFs are translated in various species [14, 16, 18, 71–74]. Longer ORFs are the most likely to be encoded [75–77]. Regulatory elements (IRES, m6A-modified conserved sites, and so on) upstream in the open reading frame mediate translation [78, 79]. The positional relationship between the ORF and the cyclization site is significant in circRNAs. In general, an ORF spanning the splicing site is the distinctive feature of circRNA-encoded peptides [80]. Websites and software used to predict ORFs are listed in Table 1.

**Table 1:** Classification of prediction methods for ORFs

Name	Characteristics	Website
CircRNADb [81]	It includes 32,914 circRNA records for human exons. Each of them has IRES sequence components, predicted ORFs, related references, and so on. The predicted ORF is usually the cross-splicing site.	http://202.195.183.4:8000/circrnadb/circRNADb.php
sORFs.org [82, 83]	It comprises > 4,374,422 sORFs from six different species and derived from multiple ribosome profiling and sequencing datasets.	http://www.sorfs.org
ORF Finder [84]	It performs six-frame translations and returns the range of each ORF and its protein translation. These may be submitted directly for BLAST similarity or COGs database searches.	https://www.ncbi.nlm.nih.gov/orffinder/
ORF Predictor [85]	It was designed to predict expressed sequence tags (EST) or cDNA sequences. Its output file consists of predicted coding DNA sequences, the start and end of the coding region, and the protein peptide sequence.	http://bioinformatics.ysu.edu/tools/OrfPredictor.html
SMS:ORF Finder [86]	It searches for newly-sequenced DNA and returns the range of each ORF and its protein translation. SMS:ORF Finder supports the entire IUPAC alphabet and several genetic codes.	http://www.bioinformatics.org/sms2/orf_find.html
CSCD [87]	It contains cancer-associated circRNA alternative splicing, expression, and translation (ORF). It is linked to UCSC which predicts potential ORFs and highlight translatable circRNAs.	http://gb.whu.edu.cn/CSCD/
PhyloCSF [88–91]	It is based on the phylogenetic analysis of multi-species genomic sequence alignments and identifies conserved protein coding regions. It requires complete ORF sequences from different species to be able to evaluate their coding probabilities.	http://compbio.mit.edu/PhyloCSF/
CircPro [92]	Its data was derived from high-throughput sequencing (RNA-Seq and Ribo-Seq). It generates a list of circRNAs and reports genomic locations, ORF lengths, junction reads from Ribo-Seq, and so on.	http://bis.zju.edu.cn/CircPro/
cORF_pipeline [93, 94]	Its output predicts sORF sequences, start and stop positions, annotation data, and so on. The longest ORF spanning the circRNA splicing site is the one most likely to encode.	https://github.com/kadenerlab/cORF_pipeline
CircBank [95]	Its output contains the ORF size, the coding potential, and circRNA conservation.	http://www.circbank.cn/index.html

#### Predictions of translation starter elements: IRES

An IRES is an RNA regulatory element that recruits ribosomes, implements ribosomal assembly and reading frame protein translation, and initiates protein translation independent of the 5' cap structure and direct translation [96–98]. An earlier study found that a circRNA constructed in vitro with an IRES recruited ribosomes and underwent translation [61]. An IRES region has been found in a wide range of viral RNAs [99, 100]. The first was detected in the small RNA virus 5' untranslated region (5′-UTR). IRES have also observed in certain eukaryotic mRNAs. About 10% of mRNAs use IRES in the 5'-UTR to recruit ribosomes [101]. Moreover, the UTR of *circ-ZNF609* may serve as an IRES facilitating *circ-ZNF609* translation in a shear-dependent manner [78]. IRES may be difficult to detect in higher eukaryotes as these organisms have highly complex genomes and cellular regulatory networks. IRES appear mainly in the 5'-UTRs upstream of the ORFs they control. However, there are exceptions. Certain IRES may be seen between the ORFs while others reside within them [102–104]. IRES sequences in cells are generally less active and efficient than those in viruses. Nevertheless, the former have good characteristics and are reliable [105, 106]. Endogenous ncRNAs with IRES may translate long polypeptide chains on a continuous ORF [107, 108]. The selective regulation of IRES-mediated translation participates in physiological and pathological processes such as cell growth, proliferation, differentiation, stress response, and apoptosis [98, 109, 110]. Websites currently used to predict IRES are listed in Table 2.

**Table 2:** IRES prediction methods

Name	Characteristics	Website
IRESite [111]	It is based on experimental data derived from 68 viruses and 115 eukaryotic cells. It furnishes information on experimental IRES fragments including their nature, function, origin, size, sequence, structure, relative position to the surrounding protein coding region, and so on.	www.iresite.org
IRESfinder [112]	It is a logit model-based forecasting tool based on 19 k-mer parameters. Its accuracy is ~80%. IRESfinder is a standalone script for Python that is applicable to high-throughput screening.	https://github.com/xiaofengsong/IRESfinder
IRESPred [113]	It predicts viral and cellular IRES via the Support Vector Machine (SVM). This predictive model integrates 35 features based on the sequence and structural properties of UTRs and their probabilities of interacting with small subunit ribosomal proteins (SSRPs). Its accuracy is ~75.75% accuracy and it had a 0.51 Matthews correlation coefficient (MCC) in blind testing.	http://bioinfo.net.in/IRESPred/
VIPS [114]	It consists of the RNAL folding, RNA Align, and pknotsRG programs. Evaluations of the UTR, IRES, and virus databases disclosed that it has superior accuracy and flexibility and can predict four different sets of IRES.	http://140.135.61.250/vips/

#### Prediction of m6A modification

The m6A modification is very common in the mRNAs and ncRNAs of higher organisms [115, 116]. The m6A modification regulates mammalian gene expression [117], as well as RNA stability, localization, shearing, and translation at the post-transcriptional level. It was recently discovered that m6A has various effects on translation [118–121]. Abnormalities in its regulatory mechanism are associated with tumorigenesis [122, 123]. Using ribosome profiling, computational prediction, and mass spectrometry, m6A-driven endogenous ncRNA translation has been found to be widespread [62, 124]. Numerous translatable endogenous circular RNAs probably contain m6A sites. To examine the ability of m6A to drive circRNA translation, an m6A-modified circRNA was constructed in vitro. The m6A reading protein YTHDF3 was tightly bound to the translation initiation factor eIF4G2. The latter promoted circRNA translation in cells [62]. Commonly used tools for screening m6A motifs are listed in Table 3.

**Table 3:** M6A prediction methods

Name	Characteristics	Website
DeepM6ASeq [125]	It is based on miCLIP-Seq data at single-base resolution and detects m6A sites. It can recognize new reader FMR1.	https://github.com/rreybeyb/DeepM6ASeq
M6APred-EL [126]	It uses position-specific k-mer nucleotide propensity, physicochemical properties, and ring function hydrogen chemical properties to optimize m6A position recognition accuracy.	http://server.malab.cn/M6APred-EL/
M6AMRFS [127]	It uses dinucleotide binary encoding and local position-specific dinucleotide frequencies to encode RNA sequences. It can identify m6A sites in multiple species.	http://server.malab.cn/M6AMRFS/
SRAMP [128]	It identifies mammalian m6A sites at single-nucleotide resolution and builds m6A site predictors. SRAMP = sequence-based RNA adenosine methylation site predictor.	http://www.cuilab.cn/sramp/
iRNA-Methyl [129]	Identifying m6A sites by incorporating the global and long-range sequence pattern information of RNA via the pseudo k-tupler nucleotide composition (PseKNC) approach.	http://lin.uestc.edu.cn/server/iRNA-Methyl
iRNA (m6A)-PseDNC [130]	It uses the Euclidean distance-based method and pseudodinucleotide composition to identify m6A sites in the *Saccharomyces cerevisiae* (yeast) genome.	http://lin-group.cn/server/iRNA (m6A)-PseDNC.php
m6Acomet [131]	It is based on the RNA co-methylation network comprising 339,158 putative gene ontology functions associated with 1,446 identified human m6A sites.	http://www.xjtlu.edu.cn/biologicalsciences/m6acomet
WHISTLE [132]	It integrates 35 genome-derived and conventional sequence-derived features. It enable direct queries of predicted RNA-methylation sites, their putative functions, and their associations with other methylation sites or genes.	http://whistle-epitranscriptome.com
pRNAm-PC [133]	It predicts m6A sites in RNA sequences based on physicochemical properties. RNA sequence samples are expressed by pseudodinucleotide composition (PseDNC).	http://www.jci-bioinfo.cn/pRNAm-PC
TargetM6A [134]	It identifies m6A sites from RNA sequences via position-specific nucleotide propensities (PSNP) and a support vector machine (SVM).	http://csbio.njust.edu.cn/bioinf/TargetM6A
AthMethPre [135]	It trains the SVM classifier using the positional flanking nucleotide sequence and the position-independent k-mer nucleotide spectrum to predict m6A sites in *Arabidopsis thaliana*.	http://bioinfo.tsinghua.edu.cn/AthMethPre/index.html
RNAMethPre [136]	It predicts m6A sites by integrating multiple mRNA features and training the SVM classifier in mammalian mRNA sequences.	http://bioinfo.tsinghua.edu.cn/RNAMethPre/index.html

#### Conservation analysis

Conserved sequences indicate potential functions and/or play important roles in cell development and regulation [137–139]. As a rule, coding region sequences are highly conserved. Evolutionary conservation (including ncRNA sequences, sORFs, and small peptide amino acid sequences) may serve as a predictor in the analysis of the coding functions of ncRNAs [71, 75, 140–142]. For conservation analysis, nucleotide- and protein-protein BLAST [23], UCSC [143, 144], and other websites may be consulted. Alternatively, software such as MegAlign [145], MEGA [146], and Clustal [147, 148] can be used.

#### Translational omics analysis

Most of the current research on ncRNA-encoded peptides is based on data analyses performed by ribosome display technology [43, 141]. The evolution of high-throughput sequencing has yielded four detection methods for translation omics analysis [149]. These include polysome profiling, ribosome immunoprecipitation/ribosome affinity purification, ribosome profiling (also known as ribo-seq), and ribosome-nascent chain complex (RNC)-seq.

Polysome profiling separates polyribosomes by sucrose density gradient centrifugation as ribosomes have high sedimentation coefficients. The rate of sedimentation during gradient centrifugation increases with the number of ribosomes bound to the mRNA. Thus, mRNAs bound to different numbers of ribosomes may be separated in solution by centrifugation. mRNAs and their active translation ORFs in the separated components are then analyzed, and the output is used to evaluate the ncRNA coding potential [78]. However, the RNA recovery for translation is low in this method and may not suffice to meet the sample size requirement for full spectrum analysis [150, 151]. Ribosome profiling is a comprehensive quantitative method to sequence the mRNA segments in ribosomes [152]. It uses low-concentration RNase to digest the RNC, degrades the mRNA fragments without ribosome coverage, and sequences and analyzes RNA fragments ~22-30 bp long. These are known as ribosome footprints or ribosome-protected fragments. The ribosome distribution and density on each transcript can be determined, as well as the starting codon, ORF location, translation pause area, and other information [153–156]. The ribosomal characteristics of hundreds of ORFs in annotated non-coding genes, as well as new peptides, may also be identified from ribo-seq data [141, 157–161]. During translation, ribosomes bind and move along the mRNA chain and gradually synthesize a protein polypeptide chain based on the codon triplet information in the mRNA template. During this process, an RNC is formed. Ribosomes and tandem mRNA precipitates may be separated by sucrose density gradient centrifugation and the mRNA further purified and separated for high-throughput sequencing, known as RNC-seq [162]. Using this method, ncRNA binding to ribosomes may also be analyzed. ncRNA can be translated into proteins in RNCs [79]. In ribosome immunoprecipitation/ribosome affinity purification, specific fusion marker proteins are used to bind ribosomal large subunits, and antibodies against these markers isolate the polymers. The mRNAs and ncRNAs are then isolated for microarray or sequencing analysis [163, 164].

#### Proteomics analysis

Proteomics can be used to discover and directly detect micropeptides encoded by ncRNAs, which in turn provides the most intuitive evidence that ncRNAs can encode small peptides. Among them, biological mass spectrometry is a common identification and analysis method for these micropeptides. Zhang et al. used immunoprecipitation combined with liquid chromatography tandem mass spectrometry (LC-MS/MS) to characterize the unique amino acid sequences encoded by *circ-FBXW7*, *circ-SHPRH*, and *circPINT*. The distinctive peptides identified in the mass spectrometry results also matched the ORF prediction results [67, 68, 79]. Commonly used software or databases that can be used for protein sequence alignment and peptide search of mass spectrometry data include UniProt [165] and Mascot daemon [166].

#### Experimental method identification

As more attention has been given to ncRNA-encoded proteins, several experimental identification methods have emerged to detect these proteins. To verify predicted reading frame expression, FLAG-labeled expression vectors constructed in vitro are imported into cells. Western blots identify distinct bands at the expected molecular weight, indicating that the artificially constructed ncRNA with the FLAG label was translated. CRISPR has also been used to knock FLAG labels into endogenous ncRNA coding regions and detect endogenous protein expression. Sucrose density gradient centrifugation, puromycin treatment, and other techniques determine the extent to which the target ncRNA recruits and binds the ribosomes in the translation machinery. Dual luciferase and other reporting assays elucidate IRES activity and predict ncRNA encoding ability [78, 167]. Overexpression and mutation experiments demonstrate the functions of each regulatory sequence and site. Use of vectors with manipulation of translational elements, such as mutated forms of the predicted IRES, m6A modification sites, or ATG start codons, may confirm whether the translation occurs as normal and the phenotype is consistent. Endogenous translation products may be identified by western blot or with specific antibodies such as those designed for unique amino acid sequences across the circRNA splicing site [67, 68] or common amino acid sequences encoded by lncRNA- and circRNA-derived transcripts [168]. In this way, the translational functions of the endogenous circRNAs may be verified, and the overexpression and knockdown of the translation products can be simulated. The proteins and polypeptides in the samples are isolated and determined by LC-MS/MS.

### Tumor-related functional peptides

Research on ncRNA-encoding proteins has been increasing in recent years. Multiple ncRNAs encode small peptides and regulate various malignant tumor phenotypes, such as cell proliferation, invasion, and metastasis. Below are certain tumor-related functional peptides known to be encoded by circRNAs and lncRNAs.

#### SHPRH-146aa

**Fig. 1:**
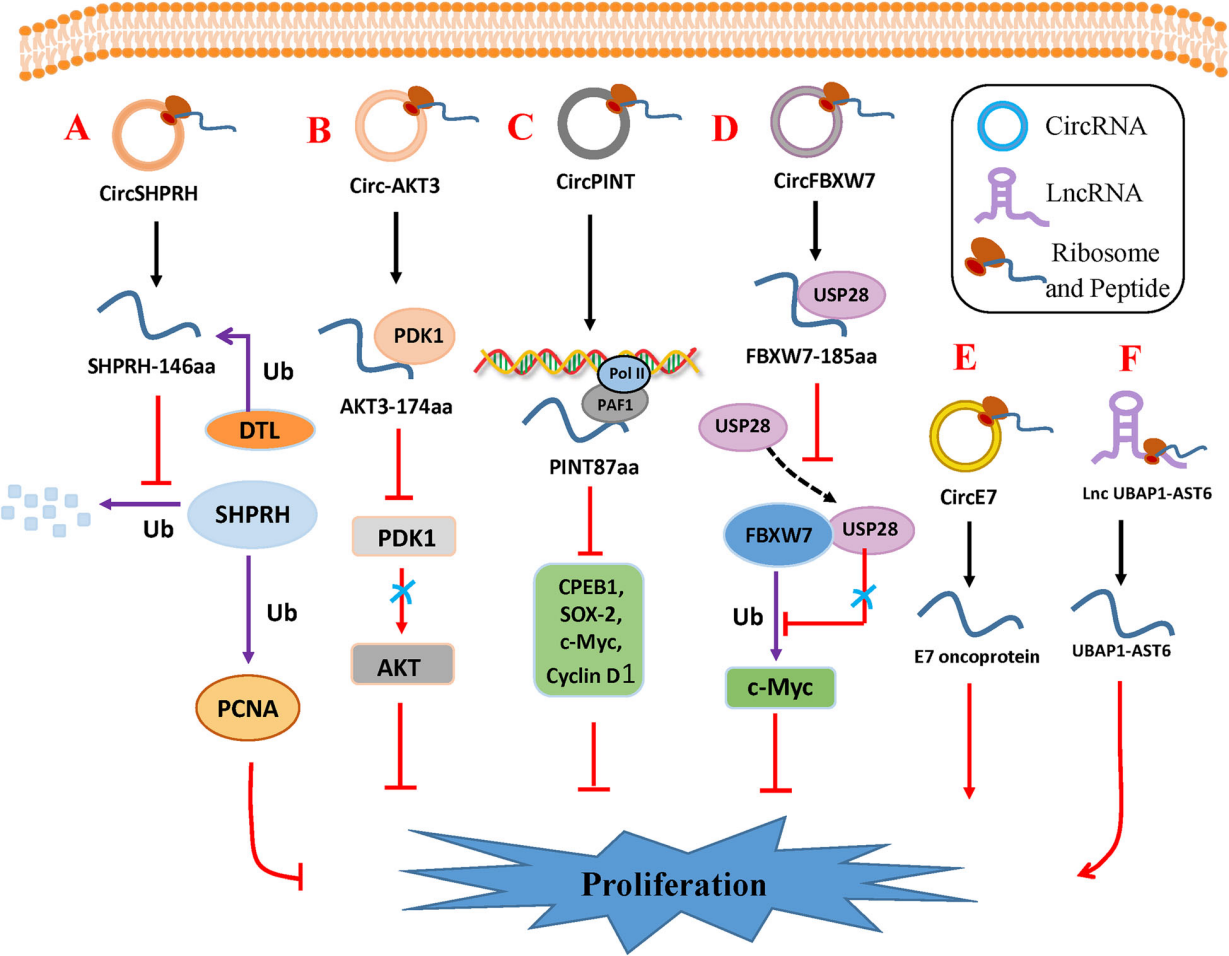
Small peptides encoded by circRNAs and lncRNAs regulate tumor proliferation. **a***CircSHPRH* encodes SHPRH-146aa, which protects full-length SHPRH from ubiquitin protease degradation. SHPRH ubiquitinates PCNA as an E3 ligase. **b***Circ-AKT3* encodes AKT3-174aa, which competitively interacts with PDK1 to negatively regulate the PI3K/Akt signaling pathway. **c***CircPINT* encodes PINT87aa, which interacts with PAF1 and inhibits transcriptional elongation of oncogenes. **d***Circ-FBXW7* encodes Fbxw7-185aa, which prevents interaction between USP28 and FBXW7a by competitively binding USP28 and destabilizing c-Myc. **e***CircE7* encodes the E7 oncoprotein, which promotes tumor proliferation. **f** The lncRNA *UBAP1-AST6* encodes UBAP1-AST6, which is a cancer-promoting factor

The circular form of the SNF2 histone linker PHD RING helicase (*SHPRH*) gene encodes the protein SHPRH-146aa. *Circ-SHPRH* and SHPRH-146aa are highly expressed in normal human brain tissue and downregulated in glioblastoma. Cyclization in *circ-SHPRH* results in the tandem stop codon UGAUGA. The entire *circ-SHPRH* is translated into a 146-aa protein by starting and stopping translation with overlapping genetic codes. An antibody against the unique amino acid sequence generated by the ORF spanning the splicing site and identification of the SHPRH-146aa amino acid sequence by LC-MS/MS confirmed that *circ-SHPRH* was translated into SHPRH-146aa. The latter participates in the development of central nervous system cancer through regulation of protein ubiquitination pathways. SHPRH-146aa overexpression in U251 and U373 glioblastoma cells reduces their malignancy and tumorigenicity in vitro and in vivo. SHPRH-146aa protects full-length SHPRH from degradation by ubiquitin proteases. It also stabilizes SHPRH as an E3 ligase by ubiquitinating proliferating cell nuclear antigen. In this manner, it inhibits cell proliferation and tumorigenicity [68, 169] (Fig. 1a).

#### AKT3-174aa

*Circ-AKT3* is formed by the cyclization of the third to seventh exons of *AKT3.* It is 524-nt long and localized mainly to the cytoplasm. When it is driven by an active IRES, *circ-AKT3* encodes a 174-aa protein, AKT3-174aa, via the overlapping start-stop codon UAAUGA. AKT3-174aa has the same amino acid sequence as residues 62–232 of AKT3. Compared with normal brain tissue, AKT3-174aa is downregulated in glioblastoma tissue. AKT3-174aa, but not *circ-AKT3*, acts as a tumor suppressor. AKT3-174aa overexpression inhibits glioblastoma cell proliferation, radiation resistance, and tumorigenicity. The PI3K/Akt pathway plays central roles in various oncogenic signaling pathways promoting glioblastoma development and progression [170, 171]. After PI3K activation, Akt is recruited to the membrane via the PH-domain and is fully activated after Thr308 and Ser473 are sequentially phosphorylated. PDK1 directly phosphorylates Akt at Thr308. This initial step is the most critical in Akt activation. The amino acid sequence of AKT3-174aa is partially identical to that of AKT3. Thus, AKT3-174aa competitively interacts with activated PDK1, inhibits Akt phosphorylation at Thr308, and negatively regulates the PI3K/Akt signaling pathway [172] (Fig. 1b).

#### PINT87aa

Zhang et al. identified certain circRNAs by performing circRNA transcriptome and RNC-RNA sequencing and bioinformatics integration analysis on normal human astrocytes and U251 glioblastoma cells. The second exon of the lncRNA *LINC-PINT* formed the circular molecule *circPINT* by self-cyclization. The latter contained an sORF and a natural IRES encoding an 87-aa polypeptide translated from endogenous *circPINT* exon 2 rather than linear *LINC-PINT*, termed PINT87aa. It is localized mainly to the nucleus, directly interacts with PAF1, regulates the PAF1/POLII complex, inhibits the transcriptional elongation of the downstream oncogenes *cpeb1, sox-2, c-Myc, cyclin D1*, and others, and inhibits the proliferation and tumorigenesis of glioblastoma and other cancer cell types [79] (Fig. 1c).

#### FBXW7-185aa

*Circ-FBXW7* may have an ORF spanning the splice site. It is highly conserved among different species and encodes a 185-aa protein driven by an IRES independently of the 5' cap translational machinery. *Circ-FBXW7* was able to be translated in human cells using a construct harboring a FLAG sequence before the ORF stop codon. The *circ-FBXW7* IRES-mut vector, which has a mutation in the IRES sequence, was transfected into U251 and U373 cells. However, cells transfected with the vector formed a circular RNA similar to *circ-FBXW7*. Therefore, it is FBXW7-185aa rather than *circ-FBXW7* that induces cell cycle arrest and hinders glioma cell proliferation. FBXW7a is the most abundant isoform of FBXW7*.* It uses c-Myc as a tumorigenesis regulator for ubiquitination-induced degradation. The de-ubiquitinating enzyme USP28 stabilizes c-Myc by binding it via interaction with the N-terminus of FBXW7a. The protein FBXW7-185aa translated by *circ-FBXW7* has a relatively higher affinity for USP28. It functions as bait and competitively inhibits USP28 from binding FBXW7a. In this way, it perturbs c-Myc stabilization induced by USP28 and shortens its half-life. FBXW7-185aa is a synergistic parental gene that encodes FBXW7a, stabilizes c-Myc, inhibits tumor cell proliferation and malignant phenotypes, and impedes malignant glioma progression [67] (Fig. 1d).

#### E7 protein

Human papillomaviruses produce the 472-nt oncogene called *CircE7* containing the entire E7 ORF. *CircE7* is modified by m6A and is localized mainly to the cytoplasm. It is closely associated with polyribosomes and may be translated to the E7 oncoprotein. The latter process is upregulated by cellular stressors such as heat shock. E7 translation was increased two- to four-fold under 42 °C heat shock. *CircE7* knockdown in CaSki cervical cancer cells reduces E7 protein levels, inhibits cancer cell proliferation and colony formation, and suppresses tumor growth and malignancy. *CircE7* is essential for E7 protein expression and transformation in CaSki cervical cancer cells both in vitro and in transplanted tumors [124] (Fig. 1e).

#### UBAP1-AST6

The protein UBAP1-AST6 is translated from a lncRNA, is localized mainly to the nucleus, and is expressed in A549 lung cancer cells. UBAP1-AST6 promotes cancer, and its overexpression significantly induces cancer cell proliferation and colony formation [173] (Fig. 1f).

#### circPPP1R12A-73aa

**Fig. 2:**
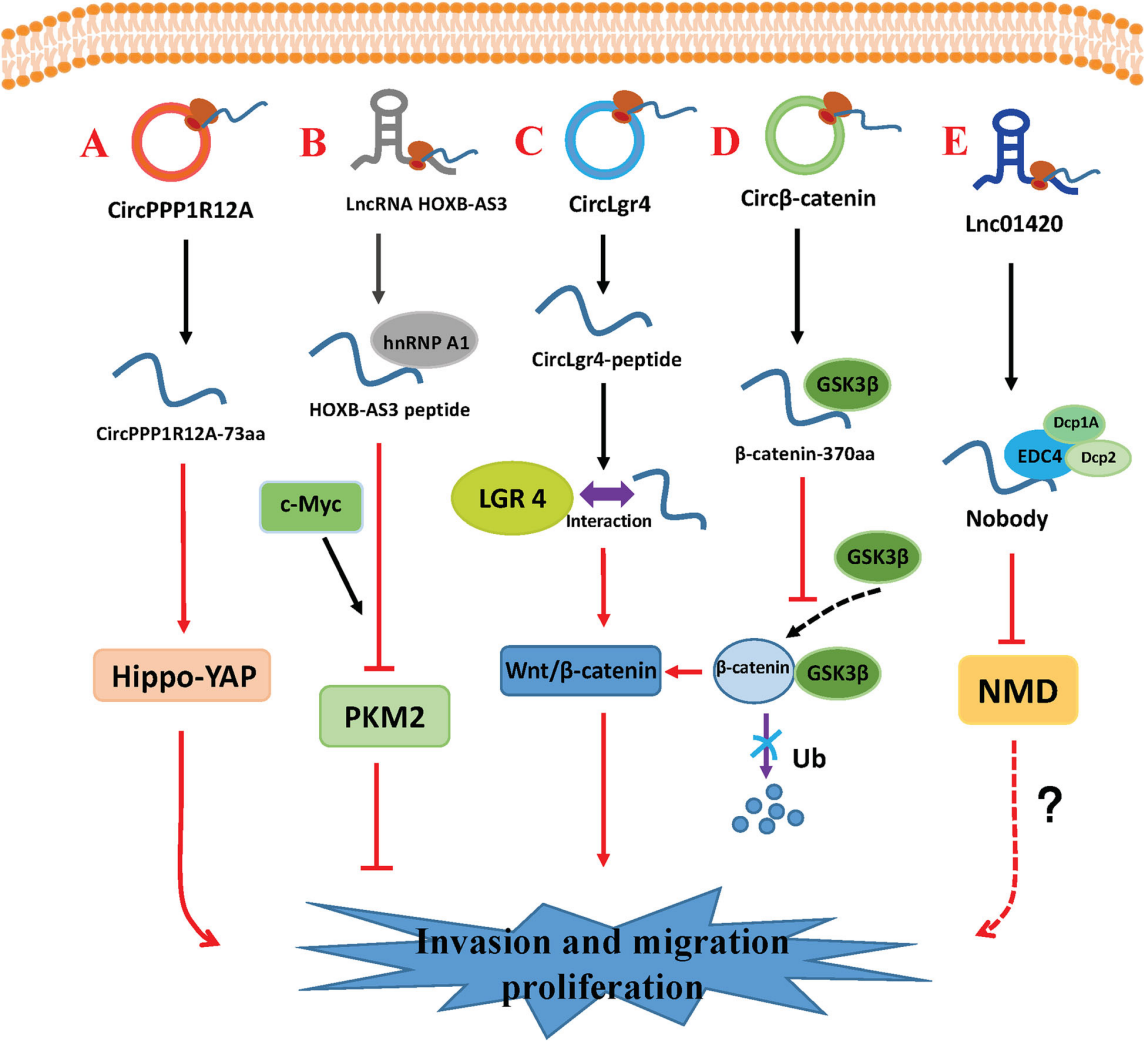
Small peptides encoded by circRNAs and lncRNAs regulate tumor invasion, metastasis, and proliferation. **a***CircPPP1R12A* encodes circPPP1R12A-73aa, which activates the Hippo-YAP signaling pathway. **b** The lncRNA *HOXB-AS3* encodes the HOXB-AS3 peptide, which competitively binds hnRNP A1 and antagonizes hnRNP A1-mediated PKM splicing regulation. **c***CircLgr4* encodes circLgr4-peptide, which interacts with LGR4 and activates the LGR4-Wnt signaling pathway. **d***Circβ-catenin* encodes β-catenin-370aa, which antagonizes GSK3β-induced β-catenin phosphorylation and ubiquitination/degradation, stabilizes full-length β-catenin, and activates the Wnt pathway. **e***LINC01420* encodes nobody, which binds EDC4 to regulate mRNA degradation. LINC01420 may promote nasopharyngeal carcinoma invasion and metastasis via this pathway

*CircPPP1R12A* is highly expressed in colon cancer tissues and serves as a prognostic marker of survival. Patients with increased *circPPP1R12A* have comparatively poorer overall survival. *CircPPP1R12A* contains a short 216-nt ORF encoding the conserved 73-aa peptide circPPP1R12A-73aa. Silencing *CircPPP1R12A* markedly inhibits colon cancer cell proliferation, migration, and invasion. Construction of the FLAG-*circPPP1R12A* overexpression vector with an initiation codon mutation ATG/ACG confirmed that it is circPPP1R12A-73aa rather than *circPPP1R12A* that plays key roles in colon cancer cell proliferation, invasion, and metastasis. The YAP1-specific inhibitor peptide 17 dramatically attenuated colon cancer cell proliferation, migration, and invasion promoted by circPPP1R12A-73aa overexpression. Induction of colon cancer growth and metastasis by circPPP1R12A-73aa was validated in vitro and in vivo by activating the Hippo-YAP signaling pathway [174] (Fig. 2a).

#### HOXB-AS3 peptide

The lncRNA *HOXB-AS3* is a tumor suppressor that is substantially downregulated in highly metastatic and primary colorectal cancer (CRC) tissues. *HOXB-AS3* binds ribosomes and encodes a highly conserved 53-aa peptide called HOXB-AS3. It is endogenous, naturally occurring, and widely expressed in various tumor tissues. HOXB-AS3 inhibits cancer cell proliferation, invasion, and metastasis and suppresses tumor growth. Colon cancer patients with low HOXB-AS3 levels generally have poor prognoses. HOXB-AS3 competitively binds arginine in the RGG motif of hnRNP A1. In this manner, it blocks hnRNP A1 binding to the pyruvate kinase M (PKM) EI9 sequence, antagonizes hnRNP A1-mediated PKM splicing regulation, and inhibits PKM 2 subtype formation and miR-18a production. HOXB-AS3 downregulates PKM2 but upregulates PKM1. PKM2 is a key regulator of aerobic glycolysis and increases lactic acid production. Therefore, HOXB-AS3 inhibits aerobic glycolysis in CRC cells. The loss of HOXB-AS3 is a key oncogenic event in CRC metabolic reprogramming [175] (Fig. 2b).

#### CircLgr4-peptide

*CircLgr4* is highly expressed in advanced CRC and is associated with poor prognosis. LGR4 is also highly expressed in colorectal tumors and activates Wnt/β-catenin signaling via ubiquitination and FZD receptor stabilization. Thus, it drives colorectal stem cell self-renewal and invasion. *CircLgr4* encodes the circLgr4-peptide, which interacts with LGR4 to activate the LGR4-Wnt signaling pathway. *CircLgr4* drives colorectal stem cell self-renewal and invasion in a manner dependent on LGR4. The circLgr4-peptide-Lgr4 axis may be used in targeted CRC therapy [176] (Fig. 2c).

#### β-catenin-370aa

*Circβ-catenin* is derived from *CTTNB1*, which encodes β-catenin, a major regulator of the Wnt pathway in liver cancer. *Circβ-catenin* is upregulated in hepatocarcinoma tissues and is localized mainly to the cytoplasm. It has an ORF and an active IRES encoding the 370-aa β-catenin isomer β-catenin-370aa. *Circβ-catenin* knockdown inhibits hepatoma cell growth and migration in vitro and in vivo, impedes tumorigenesis and metastasis, and suppresses the Wnt/β-catenin pathway. Construction of the *circβ-catenin* expression vector with an initiation codon mutation disclosed that its functionality could be attributed to its protein-coding ability rather than its non-coding property. *Circβ-catenin* knockdown had no effect on the *CTTNB1* mRNA level but significantly reduced the β-catenin protein level. β-catenin stability is closely linked to its phosphorylation state. After β-catenin is phosphorylated by GSK3β, it is ubiquitinated by the ubiquitin ligase β-TrCP and degraded by the proteasome. β-catenin-370aa, encoded by *circβ-catenin*, interacts with GSK3β and acts as a bait to block it from binding the full-length β-catenin protein. In this manner, it represses GSK3β-induced β-catenin degradation. In liver cancer, β-catenin-370aa stabilizes β-catenin by reducing its ubiquitination, activating the Wnt/β-catenin pathway, and promoting tumor growth [168] (Fig. 2d).

#### Nobody

*LINC01420* is a lncRNA that is highly expressed in nasopharyngeal carcinoma. The overall survival rate is low in patients with nasopharyngeal carcinoma presenting with elevated *LINC01420* expression. *LINC01420* knockdown significantly inhibits nasopharyngeal carcinoma cell invasion [177]. The sORF of *LINC01420/LOC550643* encodes a highly sequence-conserved microprotein named nobody. It interacts with an mRNA capping protein, directly binds EDC4, removes the 5' cap from mRNA, promotes 5'-to-3' decay, and regulates the degradation of normal and aberrant transcripts. Nobody is localized mainly to P-bodies. Its level decreases with increasing P-body number. The latter perturbs the homeostasis of endogenous cellular nonsense-mediated decay substrates. Nevertheless, the effects of this process on tumor growth, development, and metabolism are unclear [178] (Fig. 2e).

#### Other functional peptides

*LINC00961* is substantially downregulated in human non-small cell lung cancer (NSCLC). Low tissue *LINC00961* levels are associated with clinical stage, lymph node metastasis, and shorter survival time in NSCLC patients [179, 180]. *LINC00961* may also inhibit tumor progression in oral squamous and renal cell carcinoma, glioma, and other cancers [181–184]. Matsumoto et al. reported that *LINC00961* is translatable. Its encoded small peptide SPAR is localized to the late lysosome and interacts with lysosomal V-ATPase. SPAR functions upstream of Rags and the Ragulator complex and at the v-ATPase level. It induces interactions of the v-ATPase-Ragulator-Rags supercomplex. SPAR impedes lysosomal mTORC1 reuptake, inhibits mTORC1 activation by amino acid stimulation, and affects muscle regeneration [185–187]. *Circ-ZNF609* is formed from the cyclization of the second exon of *ZNF609.* It is upregulated in nasopharyngeal carcinoma, renal and breast cancer, and other cancers. *Circ-ZNF609* knockdown dramatically inhibits cancer cell proliferation, invasion, and metastasis [188–190]. Bozzoni et al. reported that *circ-ZNF609* was strongly expressed in muscle cells, highly conserved evolutionarily, and contained a 753-nt ORF. Its UTR had IRES-like activity and encoded a protein in a splicing-dependent manner. This peptide regulated myoblast proliferation [78]. MiPEP-200a and miPEP-200b, encoded by primary miRNAs (*miR-200a* and *miR-200b*), can inhibit the migration of prostate cancer cells by regulating the epithelial to mesenchymal transition of tumor cells [191].

CircRNAs, lncRNAs, and the small peptides they encode may regulate tumorigenesis. Moreover, certain ncRNAs in various species encode proteins that regulate various biological and disease processes in vivo. For example, peptides 11–32 aa long encoded by sORFs from polished rice control epidermal differentiation in *Drosophila* by modifying the transcription factor Shavenbaby [14]. Myomodulin (MLN) is a highly conserved micropeptide encoded by a 138-nt ORF in a lncRNA. MLN structurally and functionally resembles phospholipids and phosphatidylcholine and inhibits SERCA in a similar manner. In this way, MLN regulates muscle motility [15]. A muscle-specific lncRNA encodes a small 34-aa peptide, DWORF. It increases the activity of SERCA pumps which, in turn, enhance cardiac contractility during a heart attack [192]. In the *Drosophila* heart, a lncRNA (*pncr003:2L*) encodes two peptides ≤ 30-aa long that regulate calcium transport and affect muscle contraction [23]. Pauli et al. found that the short, conserved polypeptide Toddler encoded by a lncRNA in zebrafish promotes cell movement during gastrulation by activating APJ/apelin receptor signaling [193]. *Pri-miR171b* from alfalfa and *pri-miR165a* from *Arabidopsis* produce peptides that promote the accumulation of mature miRNAs and downregulate target genes regulating root development [194]. Kadener et al. reported that *circMbl*-encoded proteins are enriched in synaptosomes and modulated by starvation and FOXO [94]. Abou-Haidar et al. found a covalently closed, 220-nt circular RNA in a viroid. The translated protein was rich in basic amino acids, expressed only in RYMV-infected rice plants, and bound homologous (scRYMV) and heterologous [potato virus X] RNA [195].

## Conclusions and future perspectives

NcRNA-encoded proteins have attracted a great deal of scientific curiosity. Research has established the existence and confirmed the importance of ncRNA-encoded functional peptides. However, the assessment of ncRNA coding potential is difficult [79]. The database used to predict interspecies conservation of ORFs, IRES, and m6A in ncRNAs is incomplete, and experimental validation protocols are still under development [196]. Most circRNAs are produced by protein-encoded exons, which may overlap with their associated mRNAs and render it difficult to distinguish the source of the translation product. High-throughput analytical and detection methods such as ribosome profiling have technical challenges [149, 152]. The identification of small peptides requires specific biochemical and bioinformatics methods seldom applied in genome-wide characterization. Moreover, cell- and tissue-specific expression complicate these assays. Therefore, the actual number of translatable sORFs and their biological functions remain unknown.

Here, we reviewed the recent advances in ncRNA-encoded small peptides regulating human cancer behavior. This investigation provided new perspectives on ncRNA functions and mechanisms. Therefore, it also suggests that future research on ncRNA may be conducted in depth in several areas, including whether there are more functional peptides or proteins encoded by ncRNA, were the ncRNAs of earlier studies analyzed as RNA or were they examined for their potential coding functions, what is the mechanism of the dynamic translation of ncRNAs encoding functional peptides, do ncRNAs encoding small peptides undergo post-translational modification in a manner similar to that for mRNA, and which factors and conditions affect ncRNA translation.

In the future, functional peptides encoded by ncRNAs may be routinely applied in cancer research, therapy, diagnostics, and prognostics, due to their potential developmental value and clinical utility. NcRNAs can encode some cancer-suppressive peptides/proteins (e.g., FBXW7-185aa, SHPRH-146aa, AKT3-174aa, and PINT87aa). Researchers can deliver these peptides/proteins to tumor cells through nanoparticles or recombine them with adenovirus and inject them into patients as anti-cancer therapy [197]. Moreover, these peptides/proteins can be used with classical anticancer drugs or in combination with traditional radiotherapy and chemotherapy to enhance the effectiveness of cancer therapy. These functional peptides encoded by ncRNAs can also play an important role in tumorigenesis, which makes them potential new targets for drug development. Researchers are also attempting to rescue or strengthen the function of tumor suppressor peptides/proteins by vaccination with synthetic peptides or viral vector vaccines encoding relevant peptides sequences for cancer therapy [198]. The application of these hidden peptides/proteins encoded by ncRNAs as therapy targets in cancer is increasingly promising. Additionally, ncRNA itself can perform biological functions and act as a molecular marker or potential target. Therefore, both functional peptides and ncRNAs can be used as cancer biomarkers for clinical applications at the dual levels of transcription and translation, helping to improve the accuracy and specificity of diagnosis and treatment. In the future, the differential expression and prognostic correlation of these peptides/proteins in cancer may also be determined through more experimental analysis and clinical examination, such as the immunohistochemical analysis of paraffin sections of tumor tissues and body fluid examination.

Here, we discussed how genetic information may also be transferred from ncRNAs to proteins and that this mechanism may participate considerably in the regulation of certain biological and oncological processes. This may help us further clarify biological operating mechanisms and regularity. As functional peptides encoded by ncRNAs is a comparatively new experimental and research field, its mechanisms, functions, regulatory factors, and prospective clinical and scientific applications require and merit further investigation.

## Data Availability

Not applicable

## Abbreviations

BLAST: Basic local alignment search tool
circRNA: Circular RNA
COGs: Cluster of orthologous groups
CRC: Colorectal cancer
EST: Expressed sequence tag
GSK3β: Glycogen synthase kinase 3β
HPV: Human papillomavirus
IRES: Internal ribosome entry site
LC-MS/MS: Liquid chromatography/tandem mass spectrometry
lncRNA: long non-coding RNA
m6A: N6-methyladenosine
MCC: Matthews correlation coefficient
miRNA: microRNA
MLN: Myomodulin
ncRNA: non-coding RNA
NMD: Nonsense-mediated decay
NSCLC: Non-small cell lung cancer
ORF: Open reading frame
PCNA: Proliferating cell nuclear antigen
PDK1: Phosphoinositide-dependent kinase-1
PI3K: Phosphoinositide 3-kinase
PKM: Pyruvate kinase M
pri-miRNA: Primary microRNA transcript
PseDNC: Pseudodinucleotide composition
PseKNC: Pseudo k-tupler nucleotide composition
RFP: Ribosome footprints
RNC: Ribosome-nascent chain complex
RPF: Ribosome-protected fragments
SERCA: Sarco-endoplasmic reticulum Ca2+ adenosine triphosphatase
SHPRH: SNF2 histone linker PHD RING helicase
sORF: Short open reading frame
SPAR: Small regulatory polypeptide of amino acid response
SRAMP: Sequence-based RNA adenosine methylation site predictor
SSRP: Small subunit ribosomal proteins
SVM: Support Vector Machine
UTR: Untranslated region
